# Low levels of Cd induce persisting epigenetic modifications and acclimation mechanisms in the earthworm *Lumbricus terrestris*

**DOI:** 10.1371/journal.pone.0176047

**Published:** 2017-04-20

**Authors:** Maja Šrut, Victoria Drechsel, Martina Höckner

**Affiliations:** 1 Department of Zoology, Faculty of Science, University of Zagreb, Rooseveltov trg 6, Zagreb, Croatia; 2 Department of Ecophysiology, Institute of Zoology, University of Innsbruck, Center for Molecular Biosciences, Technikerstr. 25, A-6020 Innsbruck, Austria; Jinling Institute of Technology, CHINA

## Abstract

Toxic effects of cadmium (Cd), a common soil pollutant, are still not very well understood, particularly in regard to its epigenetic impact. Therefore, the aim of this study was to assess DNA methylation changes and their persistence in the earthworm *Lumbricus terrestris* upon chronic low dose Cd exposure using methylation sensitive amplification polymorphism (MSAP). Moreover, the biomarker response and fitness of the earthworms, as well as the expression of detoxification-related genes (metallothionein (MT) and phytochelatin synthase (PCS)) was evaluated. Low levels of Cd caused an increase in genome-wide DNA methylation, which remained partly modified, even after several months of recovery in unpolluted soil. Increased cellular stress seemed to decrease after two weeks of exposure whereas fitness parameters remained unaffected by Cd, probably as a result from the activation of detoxification mechanisms like the expression of MTs. Interestingly, even though the level of Cd exposure was very low, MT expression levels indicate the development of acclimation mechanisms. Taken together, this study demonstrates that acclimation, as well as epigenetic modifications can occur already in moderately polluted environments. In addition, these effects can have long-lasting impacts on key species of soil invertebrates and might persist long after the actual heavy metal challenge has passed.

## Introduction

Terrestrial ecosystems have been challenged in the past decades through a growing industry and agriculture and, along with that, an increase in soil pollution. Cadmium (Cd), one of the main soil pollutants, is brought into the environment mainly as a side product in the metal and mining industry, through airborne deposition, and from the usage of Cd-containing fertilizers [1]. Cd is therefore widely applied in laboratory experiments to study the effect of soil pollution on terrestrial organisms like earthworms, which are, through their soil dwelling lifestyle, well suited to examine the adverse outcome of environmental stress. However, the exact mechanisms of Cd toxicity are not well understood, particularly in invertebrates. Cd causes a range of cellular and molecular perturbations in exposed organisms and it has been associated with enzyme inhibition, generation of reactive oxygen species (ROS) and perturbation of apoptosis or cell proliferation [2–4]. In soil organisms (e.g. earthworms), Cd affects survival, growth rate, sexual maturation and reproductive success in a dose-dependent manner [5] and exhibits genotoxic effects in different earthworm species [6–12]. Upon heavy metal insult, stress response mechanisms like the expression of metallothioneins (MTs) and production of phytochelatin (PC) are activated [13,14] and are responsible for the detoxification of Cd and the binding of ROS. Those mechanisms have also been implicated in the adaptation to changing environmental conditions [15,16].

Cd has also been shown to cause genomic instability without exerting genotoxicity, occurring through epigenetic mechanisms [17,18]. It has been described that acute exposure to Cd causes inhibition of DNA methyltransferase activity (DNMTs; i.e., enzymes involved in establishing and maintaining DNA methylation patterns) resulting in a dose-dependent decrease of DNA methylation. In contrast, chronic low-dose Cd exposure has been linked to overexpression of DNMT genes as well as to an enhanced activity of DNMTs which leads to DNA hypermethylation [17,19–21].

Since epigenetic alterations can have a profound impact on gene expression, several studies have recognized the potential of metal-induced epigenetic alterations as informative factors in the risk assessment process, for instance as biomarkers of effect [22–24]. Furthermore, DNA methylation “footprints”caused by heavy metals could aid in introducing novel predictive tools for environmental quality monitoring [25].

Persistence of epigenetic alterations even after a stressor disappeared and their potential heritability might have broader consequences for the ecosystem than formerly thought. Heavy metal pollution of terrestrial ecosystems can locally be of short duration due to e.g. eluviation. However, earthworms in polluted areas might be affected for longer periods with a putative impact on following generations. Considering the epigenome as a stress memory, soil dwelling organisms might serve as good models to study the exposure history of ecosystems and environmental health. Furthermore, earthworms are adequate invertebrate models for epigenetic studies as they have high DNA methylation levels (~13%), suggesting an important regulatory role for the epigenome, which is not the case for traditionally used model invertebrates (e.g. nematodes and fruit flies) [26,27].

Taken together, it is essential to define earthworm responses to environmental pollutants with special emphasis on epigenetic (e.g. DNA methylation) changes in order to truly understand the impact of environmental soil pollution. The aim of the present study was to evaluate the impact of chronic exposure to low, environmentally relevant concentration of Cd (10 mg/kg) on earthworm fitness and reproductive success, biomarker responses (oxidative stress, DNA damage, and lipid peroxidation), detoxification-related gene expression (MT and PCS gene expression), and alterations of genome-wide DNA methylation patterns as well as their persistence. Earthworm fitness was not affected by Cd and only slight effects like oxidative stress during early stages of the exposure has been determined. The more surprising was the finding that the genome-wide methylation level changed and that we were able to detect a Cd-dependent acclimation response.

## Materials and methods

### Earthworm origin, maintenance and exposure

*L*. *terrestris*, an environmentally relevant model species, can be easily maintained, is able to thrive and reproduce under laboratory conditions, and is commercially available. Specimens of *L*. *terrestris*, originating from a single population, as well as soil used for earthworm exposure (mixture of peat and humus), were obtained from the company Wurmwelten (Germany). Adult (clitellate) earthworms were kept at 15°C in a 12/12 light/dark cycle in sterilized soil (120°C, 12 h) with a soil water content of 50% and fed weekly with horse manure (1.2 g manure per individual) for 4 weeks prior to the start of the experiments. Exposure experiments were performed under the same conditions using Cd-spiked soil at concentrations of 10 and 60 mg CdCl_2_/kg dry soil. Soil samples were taken at the beginning and at the end of the exposure period to determine the Cd content.

#### Experimental scheme

In short, earthworms were kept either in clean soil or in soil spiked with a low (10 mg/kg) Cd concentration (exposure period 1). After 12 weeks of exposure, control earthworms stayed in clean soil and earthworms from the 10 mg/kg Cd group were transferred to clean soil (exposure period 2). Subsequently, control earthworms were divided into two groups–one was kept in control soil while the other group was exposed to a high Cd concentration. Also earthworms from the 10 mg/kg Cd treatment were divided into two groups and exposed for 2 weeks to either clean soil or soil spiked with a higher cadmium concentration (exposure period 3). A subset of Cd-exposed earthworms after the exposure period 1 was left in clean soil for 7 months.

Exposures were performed in two independent experiments (Replica I, and Replica II), each in four groups consisting of 45 earthworms. Detailed information about the experimental setup and the sampling procedure is available in S1 File.

### Earthworm fitness parameters

Earthworm fitness was assessed by measuring the weight at the beginning and at the end of the exposure period as well as by assessing reproductive success (the amount of produced cocoons and their hatchability) after the exposure period. Cocoons were kept in 6 well plates on moist filter paper in the dark at 22°C and the number of hatchlings were checked daily throughout 40 days.

### Cd determination in tissue and soil

Cd concentration in tissue and soil samples was determined as previously described [28,29] with slight modifications (for details see S1 File). In short, tissue samples (n = 4 for each treatment and time point) were thawed and dried for seven days at 65°C. The samples were then digested with 65% nitric acid (Merck, Germany) for 24 hours at room temperature and for five days at 68°C.

Measurements were performed applying graphite furnace atomization using a Z-8200 Polarized Atomic Absorption Spectrophotometer (Hitachi, Austria) at 228.8 nm. Prior to every measurement, a standard curve (0, 1 ppb, 4 ppb, 7 ppb, 9 ppb, 12 ppb, and 15 ppb) was used to validate the accuracy of the method by measuring standard reference material obtained from the National Research Council Canada (TORT-2 Lobster Hepatopancreas Reference Material for tissue samples) and from the Community Bureau of Reference Belgium (CRM-320 River Sediment for soil samples). Cd concentrations in the tissue samples are presented as mean±SEM. The treatments were compared using the Mann-Whitney Rank Sum Test (p≤0.05).

### Comet assay and oxidative stress parameters

Comet assay was performed using a previously published protocol [30] with slight modifications (see S1 File).

Tissue samples for measuring oxidative stress parameters were homogenized using glass beads on a Precellys^®^24 Homogenizer (Bertin Technologies, USA) at 8.400 x g for 60 s in 1 ml of 50 mM potassium phosphate buffer (1 M KH_2_PO_4_, 1 M K_2_HPO_4_, pH 7.0 with 0.1 mM EDTA). The homogenates were centrifuged at 10.000 x g at 4°C for 12 min. The supernatant was collected and the protein content was determined using a NanoDrop 2000c (Thermo Fisher Scientific, USA). Supernatants were used for catalase (CAT) and malondialdehyde (MDA) measurements on a spectrophotometer (EnSpire® Multimode Plate Reader, Perkin Elmer, USA).

CAT activity was measured at 240 nm as previously published [31] with modifications to measure in UV transparent 96 well plates [32]. The level of lipid peroxidation was indirectly determined as the formation of MDA (absorbance measured at 600 nm), a by-product of lipid peroxidation that reacts with thiobarbituric acid (TBA) [33]. A detailed procedure on CAT and MDA measurements is available in S1 File.

All results are presented as mean±SEM. Statistical analyses were performed using Mann-Whitney Rank Sum Test (p≤0.05) in SigmaPlot 13.0

### Quantitative RealTime PCR

The expression levels of MT2 and PCS were assessed using quantitative RealTime PCR [13] as detailed in S1 File. All results are presented as mean±SEM. Statistical analyses were performed using the Mann-Whitney U-test (p≤0.05) in SigmaPlot 13.0.

### Methylation sensitive amplification polymorphism (MSAP)

Genomic DNA was extracted using the GenElute Mammalian DNA miniprep kit (Sigma-Aldrich, Germany) according to the manufacturer’s instructions. MSAP was performed following a standard protocol [34,35] with slight modifications (see S1 File).

10 samples were replicated for all primer combinations to ensure consistency between runs. Error rates and fragment filtering was calculated and performed as previously recommended [36]. Prior to fragment filtering, the mean error rate across all primer combinations was 12.21%, and the median was 10% (*N* = 1236). A conservative filtering strategy was performed to retain only fragments with error rates equal or lower than the median of the error distribution. The mean error rate across all primers after fragment filtering was 4.33% (*N* = 728). Using this data set, each replicate was analysed independently (see S1 File). MSAP profiles were analysed using the R package msap [37] and classified as either “methylation-susceptible loci” (MSL) or “non-methylation loci” (NML). NML fragments were utilized to test for genomic diversity of earthworms (Fst values) using Arlequin software V 3.5.2.2. Samples from time point 0 from the control and Cd treatment group in both replicas were considered as 4 “cohorts”. The percentage of methylation for each individual across all MSL was calculated for each time point and treatment group. Groups were statistically compared using a Student’s t-test (level of significance p≤0.05) and the results are presented as mean±SEM. Differentially methylated MSL were defined as those showing a difference of more than 10% of the average methylation of Cd_4 and/or Cd_12 groups compared to the Cd_0 group. GenAlEx 6.5 software was applied to perform a Principal Coordinates Analysis (PCoA) using differentially methylated MSL. PCoA graphs were generated from the mean binary epigenetic distance between treatment groups. Differentially methylated MSL were further analysed to determine whether methylation after 4 and 12 weeks was either increased, decreased or the same as in the Cd_0 group. In order to define persistent DNA methylation modifications, differentially methylated MSL were analysed in replica I after a 7 month recovery period. Fragments that had the same methylation pattern (increased or decreased) after the recovery as during the exposure period were defined as persistent differentially methylated MSL. Results are presented as percentages of fragments that remained altered after the recovery period.

## Results

### Earthworm fitness

For both, the control and Cd-exposed earthworms, the survival rate after 12 weeks of exposure was above 87%. Earthworm weight did not significantly change during the course of the exposure or between treatments. In both treatment groups, at the end of the exposure, on average 1 cocoon/earthworm/month was collected with a hatchability of over 90% (Table 1). During 2 weeks of exposure to 60 mg/kg Cd earthworm survival in all treatment groups was 100% and their body mass did not differ between treatments (Table 1).

**Table 1:** Earthworm fitness parameters.

Treatment	No. of earthworms (n)	Time point (weeks)	Body mass (g)	Survival rate (%)	No. cocoons/ earthworm/month	Cocoon hatching (%)
Control	86	0	4.39±0.92			
10 mg/kg Cd	87	0	4.54±0.93			
Control	78	12	4.20±0.93	87.8±17.25	1.08±0.16	92.15±1.77
10 mg/kg Cd	83	12	4.45±0.78	91.15±6.29	1.11±0.06	92.65±0.92
Control–Control	20	19	4.43±0.68	100		
Control–60 mg/kg Cd	20	19	4.32±0.50	100		
10 mg/kg Cd–Control	20	19	4.24±0.62	100		
10 mg/kg Cd–60 mg/kg Cd	20	19	4.45±0.53	100		

Fitness parameters were measured as body mass, survival rate and reproductive success (number of cocoons and their hatchability) (mean±SD from both replica are shown). Survival rate at time point 19 relates to the survival during 2 weeks of 60 mg/kg Cd exposure.

### Cd determination

Metal measurement in soil samples revealed a concentration of 10.38 mg/kg corresponding to the 10 mg/kg Cd treatment and a concentration of 60.28 mg/kg corresponding to the 60 mg/kg Cd treatment.

**Fig 1:**
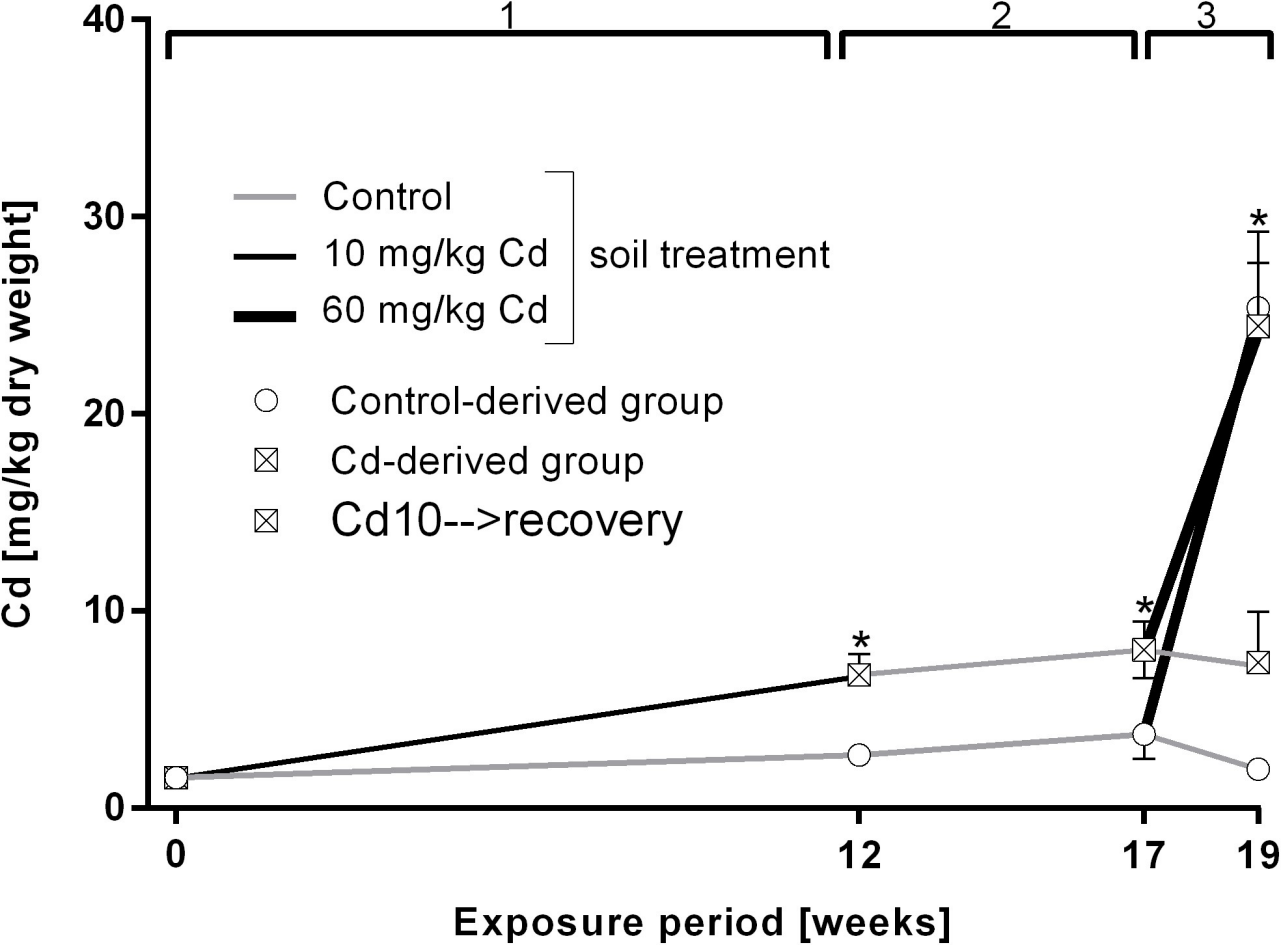
Cd accumulation in tissue of *L*. *terrestris* in control and Cadmium (Cd)-exposed individuals over 19 weeks including three exposure periods. 1: Earthworms spent 12 weeks in clean soil (grey line) or soil spiked with 10 mg/kg CdCl_2_ (black line). 2: All earthworms were kept in clean soil for five weeks (recovery). 3: Both the control- and Cd-derived group were split up into two groups, with one being transferred to clean soil (grey lines) and one being transferred to soil spiked with 60 mg/kg CdCl_2_ (thick black lines) for two weeks. The expressions "Control- and Cd-derived group" refer to the respective treatment during exposure period 1. Stars indicate significant differences to the controls (p≤0.05).

During the 12 week exposure to 10 mg/kg Cd earthworms accumulated on average 6.75 mg/kg Cd in their tissue and the level remained constant after the recovery period (Fig 1). After 2 weeks of exposure to 60 mg/kg Cd, control worms accumulated more, namely 21.6 mg/kg Cd during two weeks of high Cd exposure, whereas 10 mg/kg Cd pre-exposed earthworms (starting from higher Cd levels) accumulated 16.4 mg/kg Cd (Fig 1).

### Biomarker response

**Fig 2:**
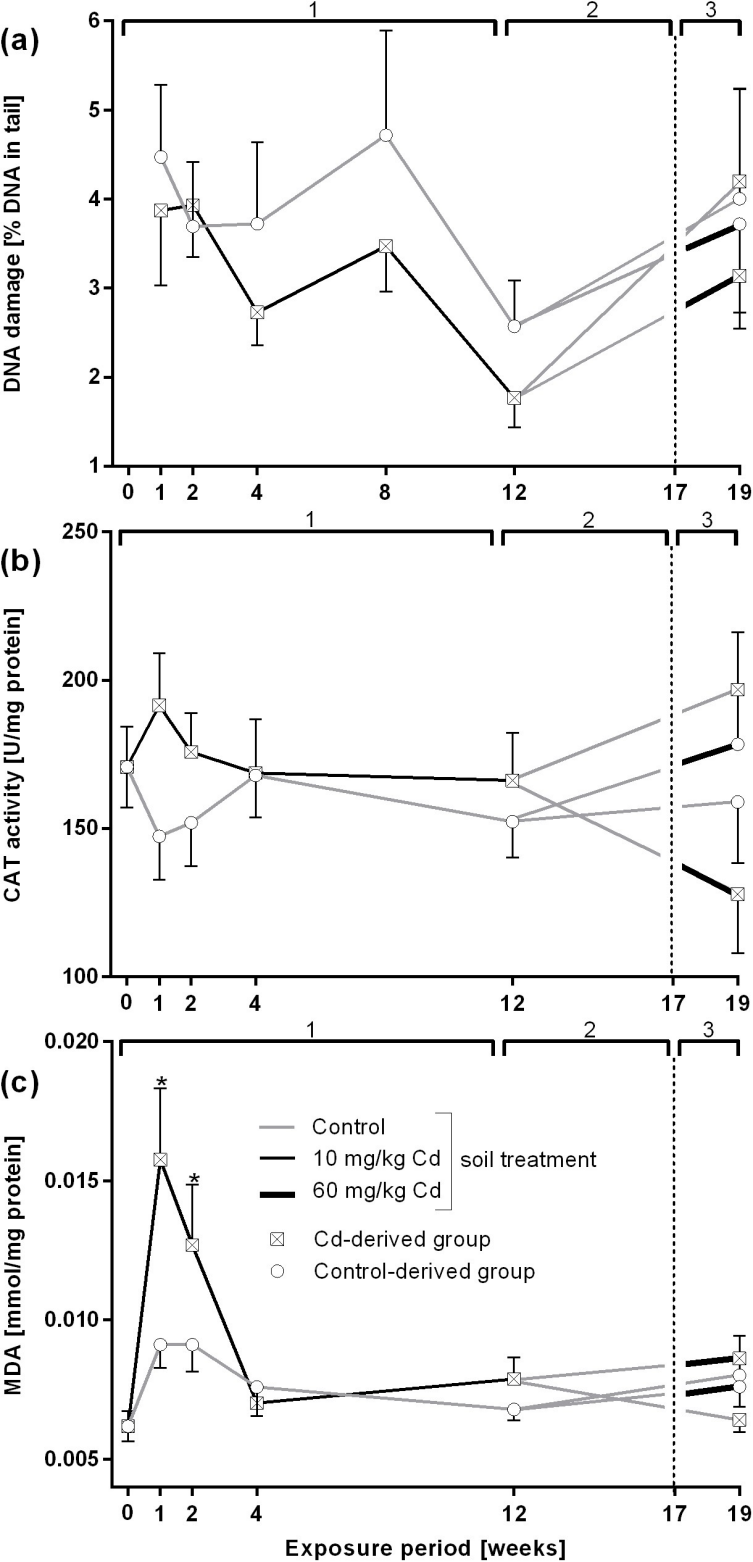
Biomarker response in *L*. *terrestris* control and Cadmium (Cd)-exposed individuals over 19 weeks including three different exposure periods. 1: Earthworms spent 12 weeks in clean soil (grey lines) or soil spiked with 10 mg/kg CdCl_2_ (black lines). 2: All earthworms were kept in clean soil for five weeks (recovery). 3: Both the control-and Cd-derived group were each split up into two groups, with one being transferred to clean soil (grey lines) and one being transferred to soil spiked with 60 mg/kg CdCl_2_ (thick black lines) for two weeks. The expressions "Control- and Cd-derived group" refer to the respective treatment during exposure period 1. (a) DNA damage measured in coelomocytes using comet assay. (b) Catalase (CAT) activity measured in tissue samples. (c) Malondialdehyde (MDA) concentrations in tissue samples. Stars indicate significant differences to the controls (p≤0.05).

The comet assay did not show significant alterations in DNA damage neither after 10 mg/kg nor after 60 mg/kg Cd exposure. However, starting at time point 4 weeks, the level of DNA damage in Cd-exposed earthworms seemed slightly lower than in controls (Fig 2A).

CAT activity was slightly increased, although not significantly, after 1 week of 10 mg/kg Cd exposure but returned to control level at 4 weeks of exposure. Exposure to 60 mg/kg Cd did not trigger any response in earthworms previously exposed to control soil. Earthworms previously exposed to 10 mg/kg Cd showed an inhibition of CAT activity at higher Cd concentrations, whereas their exposure to control soil caused an increase in CAT activity (Fig 2B).

MDA was significantly increased after 1 and 2 weeks of exposure to 10 mg/kg Cd (Fig 2C).

### Gene expression

**Fig 3:**
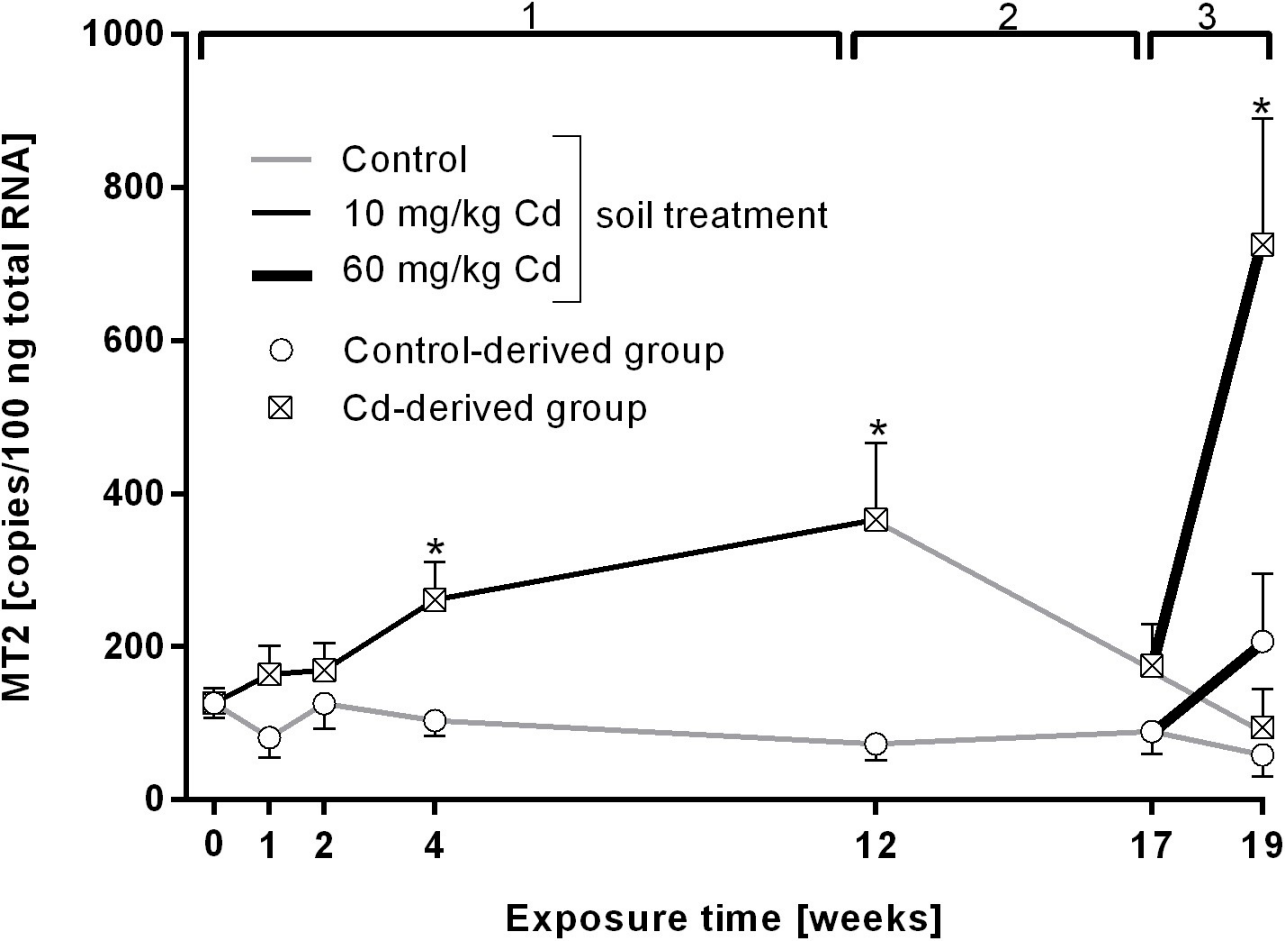
Metallothionein 2 (MT2) mRNA copy numbers in *L*. *terrestris* tissue of control and Cadmium (Cd)-exposed individuals over 19 weeks including three exposure periods. 1: Earthworms spent 12 weeks in clean soil (grey lines) or soil spiked with 10 mg/kg CdCl_2_ (black lines). 2: All earthworms were kept in clean soil for five weeks (recovery). 3: Both the control- and Cd-derived group were each split up into two groups, with one being transferred to clean soil (grey lines) and one being transferred to soil spiked with 60 mg/kg CdCl_2_ (thick black lines) for two weeks. The expressions "Control- and Cd-derived group" refer to the respective treatment during exposure period 1. Stars indicate significant differences to the controls (p≤0.05).

MT gene expression increased significantly after 4 and 12 weeks of exposure to 10 mg/kg Cd and returned to control levels after the recovery (exposure period 2) (Fig 3). Exposure to 60 mg/kg Cd revealed a significant increase of MT levels only in Cd-derived earthworms. Control-derived earthworms which were exposed to the high Cd concentration did not show a significant transcriptional induction. There was a significant correlation between Cd accumulation in the tissue and MT gene expression (Pearson correlation 0.40, p = 0.00056).

**Fig 4:**
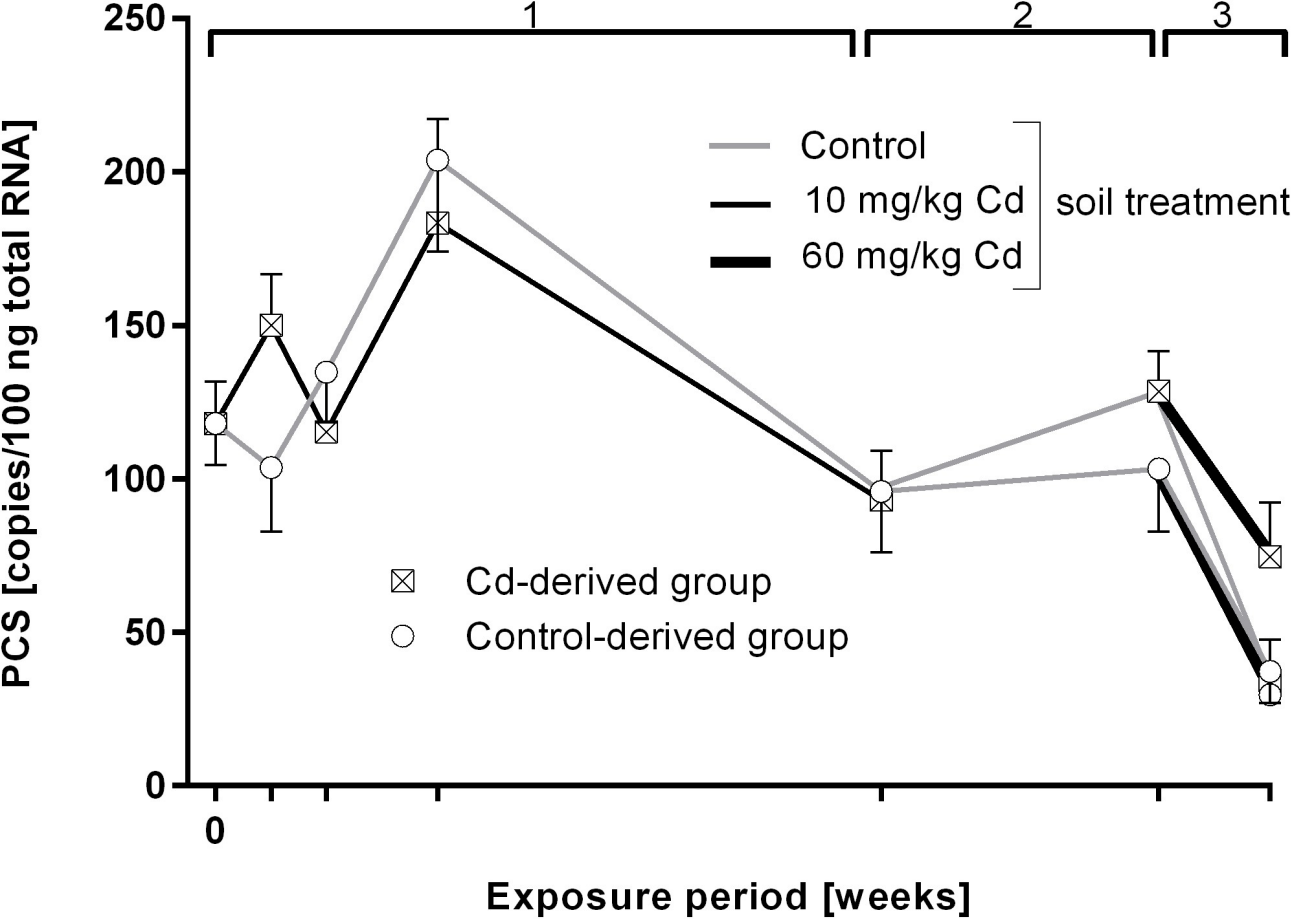
Phytochelatin synthase (PCS) mRNA copy numbers in *L*. *terrestris* tissue of control and Cadmium (Cd)-exposed individuals over 19 weeks including three exposure periods. 1: Earthworms spent 12 weeks in clean soil (grey lines) or soil spiked with 10 mg/kg CdCl_2_ (black lines). 2: All earthworms were kept in clean soil for five weeks (recovery). 3: Both the control- and Cd-derived group were each split up into two groups, with one being transferred to clean soil (grey lines) and one being transferred to soil spiked with 60 mg/kg CdCl_2_ (thick black lines) for two weeks. The expressions "Control- and Cd-derived group" refer to the respective treatment during exposure period 1. Stars indicate significant differences to the controls (p≤0.05).

PCS gene expression was not altered by Cd treatment in any of the treatment groups (Fig 4).

### DNA methylation

**Fig 5:**
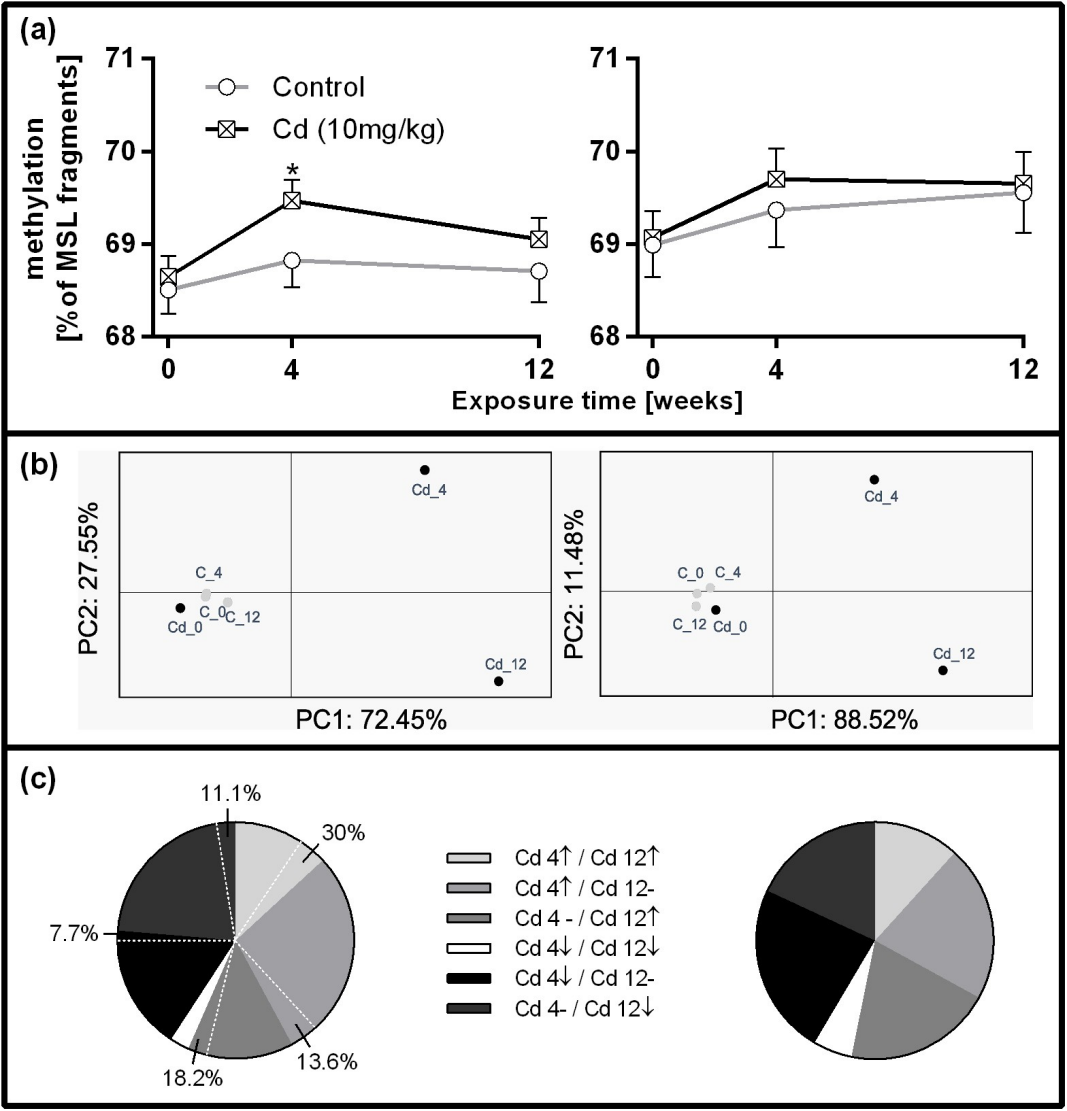
Methylation changes in *L*. *terrestris* coelomocytes in controls (C) (grey lines) and individuals exposed to soil spiked with 10 mg/kg CdCl_2_ (Cd) (black lines). Left and right panels refer to replica I and II, respectively. Only fragments where methylation did not change in the control treatment were considered for analysis. (a) Percentage of methylated fragments among all methylation states in MSL fragments in replica I (407 MSL) and replica II (463 MSL), between control and Cd-treated earthworms. (b) Principal co-ordinate analysis was performed using the data on the epigenetic distance of differentially methylated MSL between treatment groups in replica I (76 MSL) and replica II (94 MSL). (c) Type of DNA methylation change among differentially methylated MSL in replica I (76 MSL) and replica II (94 MSL). The percentages of the left pie chart indicate fragments which showed the same methylation changes after 7 months in clean soil. ↑ indicates an increase and ↓ a decrease in methylation compared to Cd 0 (earthworms used for Cd exposure sampled before the exposure start),—indicates that methylation returned to the Cd 0 level; Cd 4/Cd 12: earthworms sampled after 4 or after 12 weeks of exposure to 10 mg/kg CdCl_2_.

After fragment filtering, 541 fragments were retained in replica I and 567 in replica II. 407 polymorphic MSL and 74 polymorphic NML were determined in replica I and 463 polymorphic MSL and 50 polymorphic NML in replica II. Analysis of 26 NML overlapping between replicas revealed low genomic diversity. 99.45% of variation occurred within the population revealing an Fst value of 0.0055. The methylation state across MSL in replica I and II from all treatment groups is depicted in Table 2. Around 30–32% of the fragments were unmethylated, 4–5% showed external C methylation, 5.5–7.8% showed internal C methylation and 55.7–59% were hypermethylated (Table 2). Considering all methylation states in MSL fragments, the methylation level in Cd-treated earthworms was increased compared to the controls in replica I after 4 weeks of exposure. Replica II showed no significant difference but the same trend as replica I (Fig 5A and 5B). 18.7% of MSL in replica I (76 MSL) and 20.3% of MSL in replica II (94 MSL) were differentially methylated and discriminating between control and the Cd groups (time points 4 and 12 weeks). Based on these fragments, PCoA analysis revealed the same pattern of divergence in both replicas (Fig 5C and 5D) and PC 1 and 2 explained 100% of the epigenetic variation. The proportion and directionality of specific methylation changes was very similar between the replicas (Fig 5E and 5F). For instance 15.79% of fragments in replica I and 17.02% of fragments in replica II showed either an increase or decrease in DNA methylation, which remained consistent after 4 and 12 weeks of Cd exposure. Among differentially methylated MSL only 14 fragments overlapped between replicas. After 7 months of recovery, 11 out of 76 MSL fragments (14.5%) specific to the Cd treatment remained altered in replica I (Fig 5E).

**Table 2:** Proportion of unmethylated/methylated fragments of *L*. *terrestris* coelomocytes.

Replica I	Time point (weeks)	Unmethylated	External C methylation	Internal C methylation	Hypermethylated
Control	0	31.98±1.68	4.19±0.96	6.63±1.24	57.19±2.29
10 mg/kg Cd	0	31.78±1.64	4.82±1.32	6.82±1.00	56.58±1.69
Control	4	31.71±1.73	4.68±1.22	6.46±1.24	57.15±2.22
10 mg/kg Cd	4	30.76±1.50	4.68±1.17	6.93±0.95	57.63±1.88
Control	12	32.18±2.35	4.30±0.96	7.81±1.29	55.70±1.67
10 mg/kg Cd	12	31.55±1.36	4.26±0.93	7.66±1.22	56.53±1.63
Replica II	Time point (weeks)	Unmethylated	External C methylation	Internal C methylation	Hypermethylated
Control	0	31.01±1.68	4.51±1.04	6.26±0.90	58.22±1.81
10 mg/kg Cd	0	30.93±1.42	4.99±1.26	6.42±1.17	57.66±1.92
Control	4	30.63±2.12	4.61±1.11	5.53±1.03	59.23±1.83
10 mg/kg Cd	4	30.30±1.67	4.52±0.97	5.99±1.06	59.19±2.18
Control	12	30.44±1.94	5.04±1.27	7.05±1.10	57.47±2.00
10 mg/kg Cd	12	30.35±1.63	5.20±1.01	7.06±1.10	57.39±1.63

Proportion of unmethylated/methylated fragments across 407 MSL in replica I and 463 MSL in replica II (mean±SD) during the course of the exposure. The number of investigated earthworms in each treatment group of Replica I and II was n = 24.

## Discussion

### Biomarker response and Cd detoxification

In the present study we assessed the impact of environmentally relevant, low concentration of Cd on terrestrial ecosystems using *L*. *terrestris* as an experimental model. Changes at the organismic level as well as molecular and cellular responses and epigenetic modifications were studied over 12 weeks. A slight increase of CAT activity and MDA content was recorded within the first two weeks of Cd exposure, whereas fitness and reproductive success were not altered by Cd. Towards the end of the exposure, however, the indicators of oxidative stress (CAT and MDA) returned to the control level coinciding with the induction of MTs (at time points 4 and 12 weeks). MTs are small cysteine rich proteins, which are involved in a variety of cellular processes, inducible by Cd, and responsible for heavy metal detoxification [13]. It has already been shown that at low Cd concentrations MDA and MT are the most discriminating biomarkers [38]. Recently a putative role for PC in heavy metal detoxification has been proposed in earthworms [14,16,39]. A Cu- and Cd-induced induction of PCS, the enzyme responsible for the production of PC peptides, has been observed in *E*. *andrei* coelomocytes [40,41]. However, PCS gene expression did not change in *L*. *terrestris* tissue samples.

Interestingly, DNA damage, measured by the comet assay, did not reflect the onset of oxidative stress at early exposure stages. Rather, DNA damage seemed slightly reduced in Cd-exposed earthworms starting after 4 weeks of exposure. Reduced DNA damage has been recorded for *E*. *andrei* exposed to 100 mg/kg Cd for 14 days [42] and to Cd concentrations higher than 250 mg/kg for 28 days but only at 15°C exposure temperature [43]. In contrast to that, several studies reported a dose-dependent increase of DNA damage in *E*. *fetida* and *E*. *andrei* coelomocytes upon 7–14 days of exposure to 0.1–50 mg/kg Cd-spiked soil [10,11,44]. However, increased DNA damage has only been detected when earthworms were exposed to Cd at 20°C or at higher temperatures [10,11]. Several studies in zebrafish, rats, mice and water fleas showed that Cd rather causes the suppression of DNA repair genes [4,45–48]. Therefore, DNA damage is probably temperature- and dose-related and linked to the activation dynamics of specific detoxification mechanisms.

### Epigenetic alterations

The effects of Cd on the earthworms’ epigenome was assessed by analyzing the genome-wide DNA methylation pattern. In general, we could observe a Cd-induced increase in DNA methylation across all MSL fragments and found a similar pattern of methylation between two replicated experiments. Hypermethylation caused by low doses of Cd and prolonged exposure has been reported in various species. *Arabidopsis thaliana* cultured with 0.5–5 mg/L Cd for 16 days [49] and rat liver cells exposed to 0.5–2.5 μM Cd showed global DNA hypermethylation [17]. A similar effect was also described in human prostate cell lines during Cd-induced malignant transformation [19]. Eels exposed to 0.4 and 4 μg/L Cd were associated with a significant increase in the global CpG methylation status [50] as well as human embryo lung fibroblast cells after long-term, low-dose Cd exposure [51]. DNA methylation levels and the gene expression of DNMT1 and DNMT3a in hens’ liver and kidney were significantly elevated by Cd treatment [52]. For single genes, however, also hypo methylation has been recorded upon Cd exposure [53].

Only some of the differentially methylated fragments overlapped between our replicated experiments, which indicates that Cd affects genome-wide methylation in a stochastic way and might not be targeted to certain genomic regions. This could be due to the proposed mechanism how Cd induces DNA methylation changes. Although specific mechanisms have not been fully elucidated, emerging evidence suggests that Cd affects enzymes involved in the DNA methylation process. Animal and cell culture studies suggest that Cd alters DNMT activity [18,54–56]. Studies in plants show that Cd causes changes in DNMT activities and in coordinated expression of chromatin-related genes responsible for maintenance of various methylation patterns [57]. Furthermore, in *Posidonia oceanica* an increase in Cd-induced global genomic DNA methylation resulted from increasing activities of chromomethylase, a plant-specific DNMT [58]. This might be of paramount importance for studies implementing epigenetic markers in environmental stress assessment. It has previously been suggested that each category of compounds can have a specific DNA methylation “footprint” which can present a novel predictive monitoring tool to assess environmental quality [25]. However, this approach should be used with care and studies should be performed in several replicas, in order to assure the repeatability of methylation profiles.

A change in epigenetic marks induced by environmental stressors such as heavy metals can induce long-lasting effects. Persistent DNA methylation alterations have so far been reported in mice exposed to radiation and in primary human hepatocytes exposed to aflatoxin B1 [59,60]. Studies in *Drosophila melanogaster*, *Daphnia pulex*, *Caenorhabditis elegans*, and mammals have demonstrated even transgenerational epigenetic inheritance [61–65]. The present study shows that several fragments retained the Cd-induced methylation pattern after several months of recovery indicating the persistence of epigenetic “footprints” in earthworms.

### Development of acclimation mechanisms

In order to test whether chronic exposure to low doses of Cd leads to an acclimation response, we exposed earthworms from both the control and the Cd treatment group to either control soil or a higher Cd concentration (60 mg/kg) for 2 weeks. It is important to notice that control and Cd pre-exposed earthworms reached the same Cd levels in their tissue after the exposure to the higher Cd concentration (Fig 1). No changes in DNA damage and MDA levels were observed but interestingly, in control pre-exposed worms, CAT activity was low in both, the high Cd and the control treatment. On the other hand, in Cd pre-exposed earthworms challenged with a high Cd concentration, CAT activity was significantly decreased whereas earthworms exposed to control soil showed an increase in CAT activity (Fig 2). We found that the decrease in CAT activity was accompanied by a strong induction of MT gene expression, which can also act as radical scavenger [66,67]. A study on *E*. *andrei*, showed that low Cd caused an increase of CAT and higher doses of Cd lead to CAT inhibition [38], which could also reflect the dependency on MT expression levels.

Cd pre-exposed earthworms exhibited a fivefold higher increase in MT expression upon exposure to 60 mg/kg Cd in comparison to earthworms reared in clean soil and exposed to a high Cd concentration for the first time, although both groups exhibit the same amount of Cd in their tissues (Fig 1). We therefore suggest that the pre-exposure to low levels of Cd lead to the development of acclimation mechanisms. Similar patterns of MT induction upon laboratory exposure to Cu and Zn were noticed in *Dendrobaena octaedra* earthworms originating from metal polluted and pristine sites where metal exposure history was linked to higher induction of MT [68]. Such acclimation patterns could also be observed over several generations. For instance, the F2 generation of earthworms originating from populations with metal exposure history but raised in clean soil expressed MT more quickly and in greater quantities when exposed to metal contaminated soil than reference individuals [69]. Faster MT expression responses have already been linked to acclimatization or adaptation in the springtail *Orchesella cincta*, the earthworms *Aporrectodea tuberculate*, and *L*. *rubellus* [70–73]. Epigenetic effects have also been implicated in adaptation to changed environmental conditions, including pollution [74,75]. In human B lymphoblast cells, DNA methylation is suggested to be involved in the induction of an adaptive response to genotoxicity in cells primed with low Cd concentrations and subsequently challenged with higher Cd concentrations [75]. Acclimation to Cd could also, to a certain extent, be explained by epigenetic mechanisms. Therefore, MT regulation mechanisms, which are widely unknown in invertebrates [13], and the involvement of epigenetic factors need to be elucidated in future studies to eventually reveal the details of Cd acclimation processes in earthworms.

### Environmental relevance

*L*. *terrestris* has been proven beneficial to assess the impact of low, environmentally relevant concentrations of Cd on different levels of biological organization. The low Cd soil concentration used in this study (10 mg/kg) corresponds to the soil concentrations found in agricultural areas (8.91–11.99 mg/kg), in the vicinity of smelters (8.32 mg/kg) as well as in estuaries and deltas of polluted rivers (9.9–24.4 mg/kg) [76–79]. High Cd soil concentration used to test for acclimation (60 mg/kg) is linked to the concentrations found in more polluted environments, such as mines (up to 200 mg/kg) and lead-zinc smelters (54.5 mg/kg) [80].

Although life traits were not significantly affected and oxidative stress occurred only at early exposure stages, earthworms showed an increase in genome-wide DNA methylation levels. Some DNA methylation modifications persisted even after a prolonged recovery period. Since several authors reported the persistence of epigenetic modifications in subsequent generations [61–65], this finding is of high significance for environmental protection strategies and shows that earthworms can inform on recent and past heavy metal impact. Furthermore, several studies have reported on the potential of earthworms as adequate models for environmental epigenetic studies in heavy metal polluted soils [26,81]. The present study further proves that Cd-exposed earthworms present a very good experimental model to study the function and mechanisms of DNA methylation in invertebrates which are challenged with changing environmental conditions.

## Supporting information

S1 File Detailed description of the material and methods section. (DOCX)
