# Supplementary material for: Low levels of Cd induce persisting epigenetic modifications and acclimation mechanisms in the earthworm Lumbricus terrestris
Source: PLoS One. 2017 Apr 20;12(4):e0176047. doi: 10.1371/journal.pone.0176047 (PMC5398608; doi:10.1371/journal.pone.0176047)
Supplement: S1 File — (DOCX) [file pone.0176047.s001.docx]

**Supporting information**

**Low levels of Cd induce persisting epigenetic modifications and acclimation mechanisms in the earthworm *Lumbricus terrestris***

Maja Šrut^#*1^, Victoria Drechsel^#2^, Martina Höckner^*2^

^1^Department of Zoology, Faculty of Science, University of Zagreb, Rooseveltov trg 6, 10000 Zagreb, Croatia

^2^Department of Ecophysiology, Institute of Zoology, University of Innsbruck, Center for Molecular Biosciences, Technikerstr. 25, A-6020 Innsbruck, Austria

^#^ Authors contributed equally

^*^ Correspondence: Martina Höckner, martina.hoeckner@uibk.ac.at

Maja Šrut, msrut@biol.pmf.hr

**Content of supporting information**

A detailed description of the materials and methods section is provided.

**Materials and methods**

**Exposure and sampling**

Exposures were performed in two independent replicas each consisting of four groups as shown in Table A. Two groups in each replica were used for the 10 mg/kg Cd treatment and two for the control. The group used for biomarkers (BM), gene expression (GE) analysis and Cd tissue quantification (Cd) consisted of 45 earthworms, same as the group used for MSAP.

**Table A. Schematic representation of the experimental setup.**

|  | Replica I | | | | Replica II | | | |
| --- | --- | --- | --- | --- | --- | --- | --- | --- |
| Treatment | Control | | 10 mg/kg Cd | | Control | | 10 mg/kg Cd | |
| n | 45 | 45 | 45 | 45 | 45 | 45 | 45 | 45 |
| Group | BM, GE, Cd | Fitness  MSAP | BM, GE, Cd | Fitness  MSAP | BM, GE, Cd | Fitness  MSAP | BM, GE, Cd | Fitness  MSAP |

BM: biomarkers (comet assay, catalase, malondialdehyde), GE: gene expression (metallothionein, phytochelatin synthase), Cd: cadmium tissue quantification, MSAP: methylation sensitive amplification polymorphism. Fitness: Earthworms used for determining weight, reproduction, and hatchability.

Earthworms were exposed in 80 g dry soil/earthworm spiked with CdCl_2_ for Cd exposed group. In BM, GE, and Cd group 8 earthworms were randomly sampled at specific time points (0, 1, 2, 4 and 12 weeks of exposure). For non-invasive coelomocyte extraction, earthworms were rinsed in cold phosphate buffered saline (PBS), placed in a petri dish containing 7 ml PBS and stimulated with 9 V electric current resulting in extrusion of coelomocytes through the dorsal pores. The suspension was then used for Comet assay. Starting from the most distal part, 3 tissue pieces (app 1.5 cm) from each earthworm were sampled for gene expression analysis, enzymatic activity and Cd quantification. For specific analyses the same tissue section was used. For gene expression measurements the tissue was stored in 1 ml absolute EtOH (Merck, Germany) at -20°C until further use [1]. Tissue for enzyme assays and Cd quantification was flash frozen in liquid nitrogen and stored at -80°C. The second group of earthworms was used for the assessment of fitness and DNA methylation analysis. Coelomocytes from 24 randomly collected earthworms were non-invasively sampled at time points 0, 4 and 12 weeks. The coelomocyte suspension was centrifuged at 4500 x g for 5 min, the pellet was flash frozen in liquid nitrogen and stored at -80°C until used for DNA extraction. Earthworm fitness was assessed by measuring the weight at the beginning and at the end of the exposure as well as by assessing reproductive success (the amount of produced cocoons and their hatchability) after the exposure period. Cocoons were kept in 6 well plates on moist filter paper in the dark at 22°C and the number of hatchlings were checked daily throughout 40 days. After the low Cd exposure period, earthworms were transferred to the clean soil for 5 weeks (recovery period). Upon the recovery, the earthworms from the BM, GE, Cd group were used for gene expression analyses whereas the ones from the MSAP group (from both control and Cd treatment) were divided into two groups (control and 60 mg/kg Cd; 10 earthworms per group) and exposed for 2 weeks in order to test for the occurrence of acclimation mechanisms. After the 2 week exposure, coelomocytes and tissue samples were collected as described. A subset of earthworms from replica I was left in clean soil for 7 months after the end of the low Cd exposure period in order to monitor persistent DNA methylation changes. After the 7 month recovery period earthworm coelomocytes were sampled as previously described.

**Cd determination in tissue and soil**

The Cd concentration in tissue samples was determined in four individuals from each replica at time points 0, 12, 17 and 19 weeks in both control and Cd-treated earthworms. Tissue was thawed and dried in screw-capped polypropylene tubes (Greiner Bio-One, Austria) at 65°C for seven days. 2 ml of 65% nitric acid (Merck, Germany) in distilled water (1:1) was subsequently added and the tissue samples were digested for 24 hours at room temperature and left for five days at 68°C. Digested samples were diluted with deionised water to a final volume of 11 ml. 20 μl were used for the measurement.

The Cd soil content was determined in control and Cd-spiked soil sampled at time points 0, 12 and 19 from both replica. Each sample was measured in 5 technical replicates. 1 g of air dried soil (20 hours at room temperature) was homogenized using a mortar and pestle and covered with 10 ml 1M HNO_3_ (Merck, Germany). Samples were shaken (Infors HT Ecoctron, Switzerland) for 4 hours at 200 rpm at 25°C, centrifuged for 10 min at 1.600 x g and filtered using synthetic filter wadding (Sera Aquaristik, Germany). 20 μl of the filtrate were used for the measurement.

**Comet assay and oxidative stress parameters**

For the comet assay, 50 μl aliquots of the coelomocyte suspension in PBS with 0.8% LMP (low melting point) agarose (Sigma Aldrich, USA) were placed on microscope slides pre-coated with 1% NMP (normal melting point) agarose (Sigma Aldrich, USA). Accordingly, a third layer of 0.5% LMP agarose was added. After 1 h in lysis buffer (2.5 M NaCl, 100 mM EDTA, 10 mM TrisHCl, 10% DMSO, 1%Triton X-100; pH 10) at 4°C, slides were covered with denaturation buffer (300 mM NaOH, 1 mM EDTA) for 20 min and electrophoresis was performed in the same buffer at 0.4 Vcm^-1^ and 300 mA for 30 min at 4°C. After neutralization in 0.4 M TrisHCl buffer, slides were fixed in methanol:acetic acid (3:1) and stored in the dark at room temperature. The slides were rehydrated, stained with 4% GelRedTM (Biotium, USA), and examined using an Axiovert 100 M microscope (Zeiss, Germany). On every slide at least 100 nuclei were analyzed and the extent of DNA migration was determined as percentage of DNA in tail (% tDNA) using the image analysis system CASP [2].

Catalase (CAT) and malondialdehyde (MDA) measurements were performed using a spectrophotometer (EnSpire® Multimode Plate Reader, Perkin Elmer, USA). CAT activity was assayed by measuring the decrease in absorbance at 240 nm each 10 s during 330 s. The 250 µl reaction mixture consisted of 50 mM potassium phosphate buffer (pH 7.0), 10 mM H_2_O_2_ and 12 µl sample (200 x dilution). Each sample was measured in triplicates and catalase activity was expressed as U CAT /mg protein (using the molar extinction coefficient of H_2_O_2_, ε = 40 M-1 cm-1 and path length for 96 well plate p = 0.5 cm). The mean value of decrease in absorbance within 5 minutes was calculated and used for statistical analysis.

For MDA determination 150 μl undiluted homogenate were mixed with 450 μl 10% cold trichloroacetic acid (Sigma Aldrich, USA) and centrifuged at 10.000 x g at 4°C for 15 min to precipitate proteins. Supernatant was divided in 2 glass tubes (280 μl) and mixed with the same amount of 0.7% TBA (Sigma Aldrich, USA). The solution was incubated for 15 min at 95°C and placed on ice for 5 min. The absorbance was measured in 96 well plates in a total volume of 250 μl at 532 nm. Unspecific turbidity was corrected by subtracting the absorbance at 600 nm. MDA activity was measured in four technical replicates and expressed as nmol MDA/mg protein using the molar extinction coefficient of MDA ε = 155 mM-1 cm-1 and path length p = 0.5 cm.

**Quantitative RealTime PCR**

Tissue samples were homogenized (Ultra Turrax T25, IKA Labortechnik, Germany) in 500 µl TRIZOL^®^ reagent and RNA was extracted according to the manufacturer´s instructions (Life Technologies, USA). After DNAse I digestion (Thermo Fisher Scientific, USA) the quality of extracted RNA was assessed by electrophoresis on a 1.5% agarose gel confirming the integrity of rRNA bands. The concentration of RNA was measured in triplicates using the RiboGreen^®^ assay (Molecular Probes, USA) on a plate reader (Victor X4; Perkin Elmer, USA). An amount of 450 ng total RNA was used for cDNA transcription using RevertAid™ H Minus Reverse Transcriptase (Thermo Fisher Scientific, USA) with hexamer primers (Thermo Fisher Scientific, USA).

A standard curve for MT2 and PCS quantification was prepared by a series of dilutions of known template concentrations and a primer matrix was run to determine the optimal primer concentrations. Quantification of RNA copy number was performed on a 7500 RealTime PCR system (Applied Biosystems, USA). MT2 (GenBank: AJ010263.1) primer sequences were: 5’-TGCAGGTGTCCAAAAGATGA-3’ (forward) and 5’-ATCAGCACAGCAAAGCTTCTTG-3’ (reverse). The sequences of the PCS primers were: 5´- TCATGGTCCTGAACACG-3´ (forward) and 5´- CACAAGTTGCCGAAACTC-3´ (reverse). The *L. terrestris* PCS sequence was generated using following primers: forward 5’-GGATAACGTTCGACCAGTT-3’ and reverse 5’-AGAGAAGTGTCCGCCTCC-3’ derived from *L. rubellus* pcs-1b sequence (GenBank: KC981075.1) using Titanium Taq DNA polymerase (Clontech, USA) and standard PCR conditions. Each reaction contained 10 µl Power SYBR^®^ Green PCR Master Mix (Applied Biosystems, USA), 8 µl Primer mix (2 µl 10 x BSA, 2 µl forward primer (9 µM for both MT2 and PCS), 2 µl reverse primer (3 µM for MT2 and 9 µM for PCS), 2µl H_2_O) and 2µl of cDNA template. The reaction was amplified under the following conditions: 50°C for 2 min; 95°C for 10 min; 40 repeats of 95°C for 15 s and 60°C for 1 min. Each sample was measured in triplicates and the absolute copy number of MT2 and PCS per 100 ng RNA was determined according to the standard curves and a primer efficiency of 100% as previously described [3].

**Methylation sensitive amplification polymorphism (MSAP)**

Restriction digestion and adapter ligation of 250 ng genomic DNA was performed simultaneously at 25°C for 15 h in a total volume of 25 μl using 5 U of EcoRI (Thermo Fisher Scientific, USA) and 5 U of MspI (or HpaII) (Thermo Fisher Scientific, USA). 1.5 U T4 DNA ligase (Thermo Fisher Scientific, USA) was used for the ligation of 5 pmol EcoRI and 50 pmol MspI/HpaII double stranded nucleotide adapters. The restriction ligation mixture was diluted with nuclease-free water to 2.5 ng/μl and used as a template in the pre-amplification reaction with DNA primers which are complementary to the cores of the EcoRI and MspI/HpaII adapters. Pre-selective and selective amplifications were performed with a MyCycler thermal cycler (Bio-Rad, USA). 20 μl pre-amplification mixture contained 4 μl of the diluted restriction ligation mixture, 0.2 mM of dNTPs (Thermo Fisher Scientific, USA), 0.6 μM of each primer, 1 x Titanium Taq DNA polymerase buffer and 1 x Titanium Taq DNA polymerase (Clontech, USA). After 1 min at 95°C, DNA fragments were amplified for 20 cycles under the following conditions: 20 s at 94°C, 30 s at 56°C and 2 min at 72°C. After the final elongation step for 30 min at 60°C, the pre-amplification product was diluted 1:20 with nuclease-free water and used for selective amplification. This procedure was performed using the EcoRI-MspI/HpaII primer pairs Eco-AAG, Mse/Hpa-TCC, Mse/Hpa-TAG and Eco-ACT, Mse/Hpa-TAC, Mse/Hpa-TAG. The Msp/Hpa primers were end labelled with Hex or 6-Fam (Microsynth, Switzerland). The final amplification mixture (20 μl) contained 3 μl of the diluted pre-amplification mixture, 0.2 mM of dNTPs (Thermo Fisher Scientific, USA), 0.05 μM of the EcoRI primer, 0.25 μM of the MspI/HpaII labeled primer, 1 x Titanium Taq DNA polymerase buffer and 1 x Titanium Taq DNA polymerase (Clontech, USA). After 2 min at 94°C, the DNA fragments were amplified for 30 cycles under the following conditions: 20 s at 94°C; 30 s at 66°C for the first cycle, followed by a decrease of 1°C per cycle for 10 cycles, 56°C for the remaining 20 cycles and 2 min at 72°C. A final extension step was performed for 30 min at 60°C. The DNA fragments were detected using an automatic GeneScan ABI3130 apparatus from Macrogen Inc. (Korea). The presence or absence of fragments was scored on chromatograms using GeneMapper Genotyping Software 1.5. All fragments between 50 and 400 bp and passing the threshold of 100 RFU were scored.

Upon fragment filtering, rare alleles, e.g. those differing only in one individual, were deleted from the data set. For each replica, control samples at time points 0, 4 and 12 weeks were compared and fragments showing temporal methylation changes between these time points were eliminated from the data set in order to observe methylation changes only in those fragments responsive to Cd treatment. Fragments differing in more than 15% of total methylation between control groups were eliminated from further analyses. MSAP profiles were analysed using the R package msap [4]. Every fragment was scored as follows: non-methylated state if present in both EcoRI-HpaII and EcoRI-MspI products (1/1); methylated state if present in either EcoRI-HpaII (1/0) or EcoRI-MspI (0/1) products (either internal cytosine methylation (0/1) or hemimethylation (1/0)); hyper-methylation of the target if absent from both EcoRI-HpaII and EcoRI-MspI products (0/0). Since the methylation changes in the same pool of earthworms were monitored throughout the exposure period, the state 0/0 was considered rather as a result of hyper-methylation of the restricted targets than genetic differences (i.e. mutation) as previously suggested [4]. Fragments were classified as “methylation-susceptible loci” (MSL) if the observed proportion of methylated scores (1/0, 0/1 and 0/0) exceeded a 5% threshold, and “non-methylation fragments” (NML). Only fragments showing a polymorphism, with at least two occurrences of each methylation state were used for subsequent analyses [5].

**References**

1. Tischler A, Egg M, Füreder L. Ethanol: A simple and effective RNA-preservation for freshwater insects living in remote habitats. Limnol Oceanogr Methods. 2016;14: 186–195. doi:10.1002/lom3.10079

2. Koñca K, Lankoff a, Banasik a, Lisowska H, Kuszewski T, GóŸdŸ S, et al. A cross platform public domain PC image analysis program for the comet assay. Mutat Res. 2003;534: 15–20.

3. Pérez LM, Fittipaldi M, Adrados B, Morató J, Codony F. Error estimation in environmental DNA targets quantification due to PCR efficiencies differences between real samples and standards. Folia Microbiol (Praha). 2013;58: 657–662. doi:10.1007/s12223-013-0255-5

4. Pérez-Figueroa A. Msap: a Tool for the Statistical Analysis of Methylation-Sensitive Amplified Polymorphism Data. Mol Ecol Resour. 2013;13: 522–527. doi:10.1111/1755-0998.12064

5. Herrera CM, Bazaga P. Epigenetic differentiation and relationship to adaptive genetic divergence in discrete populations of the violet Viola cazorlensis. New Phytol. 2010;187: 867–876. doi:10.1111/j.1469-8137.2010.03298.x
